# Immunopathogenesis and Virus–Host Interactions of Enterovirus 71 in Patients with Hand, Foot and Mouth Disease

**DOI:** 10.3389/fmicb.2017.02249

**Published:** 2017-11-28

**Authors:** Jonathan A. Cox, Julian A. Hiscox, Tom Solomon, Mong-How Ooi, Lisa F. P. Ng

**Affiliations:** ^1^Institute of Infection and Global Health, University of Liverpool, Liverpool, United Kingdom; ^2^Singapore Immunology Network, Agency for Science, Technology and Research, Singapore, Singapore; ^3^NIHR Health Protection Research Unit in Emerging and Zoonotic Infections, Liverpool, United Kingdom; ^4^Walton Centre NHS Foundation Trust, Liverpool, United Kingdom; ^5^Institute of Health and Community Medicine, Universiti Malaysia Sarawak, Samarahan, Malaysia; ^6^Department of Paediatrics, Sarawak General Hospital, Kuching, Malaysia

**Keywords:** Enterovirus 71, EV71, hand, foot, and mouth disease, pathogenesis, immune response, host interactions

## Abstract

Enterovirus 71 (EV71) is a global infectious disease that affects millions of people. The virus is the main etiological agent for hand, foot, and mouth disease with outbreaks and epidemics being reported globally. Infection can cause severe neurological, cardiac, and respiratory problems in children under the age of 5. Despite on-going efforts, little is known about the pathogenesis of EV71, how the host immune system responds to the virus and the molecular mechanisms behind these responses. Moreover, current animal models remain limited, because they do not recapitulate similar disease patterns and symptoms observed in humans. In this review the role of the host–viral interactions of EV71 are discussed together with the various models available to examine: how EV71 utilizes its proteins to cleave host factors and proteins, aiding virus replication; how EV71 uses its own viral proteins to disrupt host immune responses and aid in its immune evasion. These discoveries along with others, such as the EV71 crystal structure, have provided possible targets for treatment and drug interventions.

## Background

**FIGURE 1:**
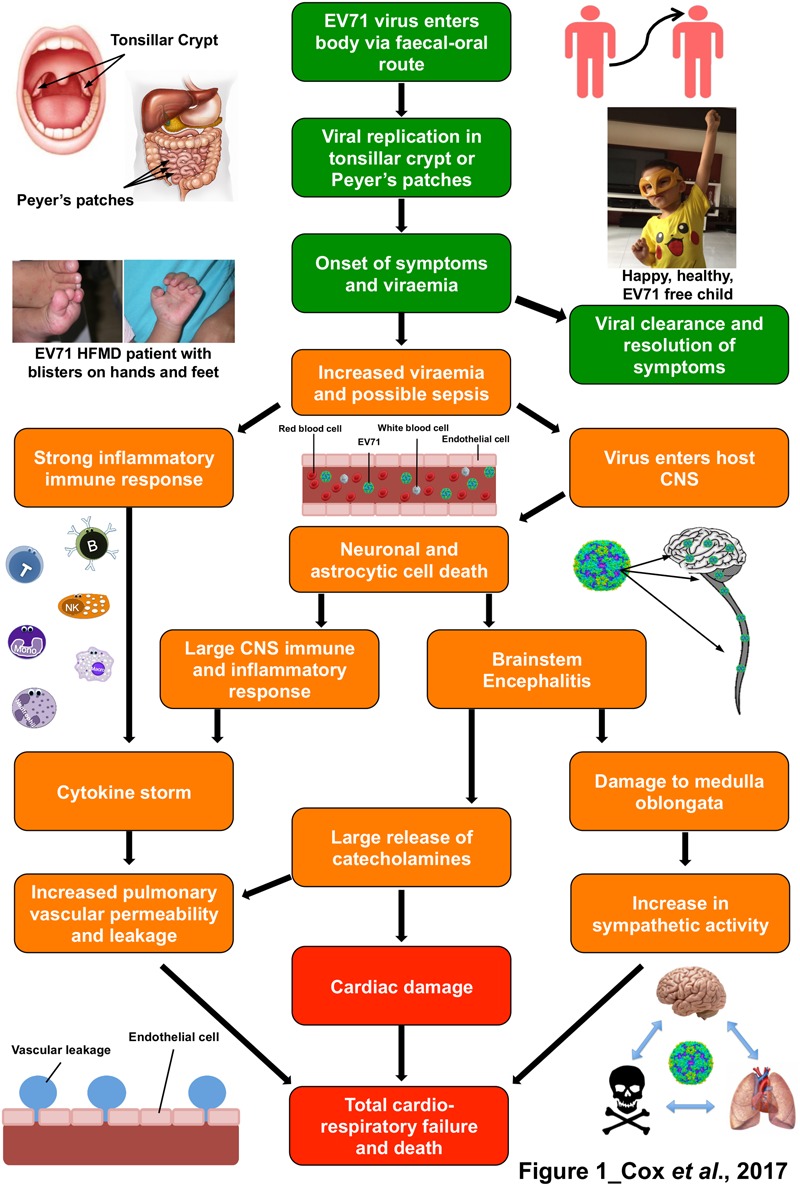
The immunopathophysiology of Enterovirus 71 (EV71) and the symptoms it leads to. Flow diagram representing the pathogenesis and immune response to an EV71 infection. 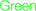 represents outcomes with mild symptoms that are usually resolved in 7–10 days, including photos of patient with hand, foot and mouth disease symptoms (Left) and a healthy child (Right). 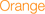 shows pathophysiology and host responses which lead to moderate to severe outcomes and 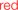 symbolizes severe to fatal outcomes. The photographs in this figure were published with consent from the parent/legal guardians of the children.

Hand, foot, and mouth disease (HFMD) is a common infection caused by a number of viruses, commonly from the *Picornaviridae* family, and notably Enterovirus 71 (EV71), coxsackie A6 (CA6) and coxsackie A16 (CA16) (Yan et al., 2001; Lo et al., 2011). HFMD predominantly affects young children, however, older children and adults can also be affected (Ooi et al., 2010; Second et al., 2017). The main symptoms of the disease are fever, and blisters on the hands, feet, and mouth. Other usual clinical signs of HFMD include, nausea, vomiting, sore throat, fatigue, malaise, loss of appetite, and irritability. About 3 to 5 days after exposure to the virus, flat, red, or discolored bumps appear, almost rash-like, on the skin around the hands, feet mouth and buttocks of the patient. These can often blister and become vesicular sores (Chang et al., 1999a). This rash is rarely itchy for infants, but it can be extremely itchy for an adult with the disease. The disease, whilst quite infectious, is normally self-limiting and symptoms usually disappear 7 to 10 days after disease onset (**Figure 1**; Chang et al., 1999a,b; Chan et al., 2000; McMinn et al., 2001; Shah et al., 2003; Sutton-Hayes et al., 2006). Although HFMD is typically a mild illness, severe complications can sometimes occur. These include encephalitis, meningitis, acute flaccid paralysis, cardiorespiratory failure and can be fatal (**Figure 1**; Crabol et al., 2017).

The coverage of HFMD, with the exception of Polar regions, is global in distribution (Ferson and Bell, 1991; Bendig and Fleming, 1996; Castro et al., 2005; Tu et al., 2007; Rabenau et al., 2010; Bracho et al., 2011; Flett et al., 2012; Mirand et al., 2012; Kar et al., 2013; Machain-Williams et al., 2014). The disease is most prevalent in the Asian–Pacific region where it has been endemic since the 1990’s; and has subsequently caused large-scale epidemics every few years (Ho et al., 1999; Komatsu et al., 1999; McMinn et al., 1999; Fujimoto et al., 2002; Lin et al., 2003b; Tu et al., 2007; Sazaly et al., 2009; Kim, 2010; Wu et al., 2010; Tan et al., 2011). Unfortunately, there are no specific anti-viral treatments for any of the viruses that cause HFMD. Therefore the treatment strategies for mild HFMD consist of palliative care including rehydration, analgesics for painful blisters and anti-inflammatories to reduce swelling (Wang et al., 1999; Ooi et al., 2007; Wang and Liu, 2009). There is also no specific treatment of more severe HFMD that can be complicated by cardiorespiratory collapse. Key treatments are mechanical ventilation and inotropic support. Empirical treatments such as IVIG and continuous renal replacement therapy have been used in recent Asian outbreaks, but there are largely anecdotal (Wang et al., 1999; Ooi et al., 2007).

## Epidemiology and Spread

Hand, foot, and mouth disease usually affects children between the ages of 6 months to 5 years old, as this is the time when they no longer receive the benefits of passively transferred maternal antibodies. These antibodies can aid in protecting them from the etiological agents, i.e., EV71 that cause the disease, when their own immune system is not fully developed to fight the virus on its own (Gantt et al., 2013). After the first encounter with the virus the child should start producing antibodies against the viral agent. These antibodies may offer a cross protective effect the next time the child encounters a HFMD causing virus (Mizuta et al., 2009; Huang et al., 2013; Zhang et al., 2014).

The main etiological agents of HFMD are the coxsackievirus A16 (CA16) and Enterovirus 71 (EV71), both of which are from the *Enterovirus A* species of the Enterovirus genus of the *Picornaviridae* family (AbuBakar et al., 1999; Tee et al., 2010; Zhuang et al., 2015). Other viruses such as coxsackie A5, A6, A9, A10, B2, and B5 have also been reported to cause the disease (Flewett et al., 1963; Hughes and Roberts, 1972; Lindenbaum et al., 1975; Ang et al., 2009; Hu et al., 2011; Flett et al., 2012; Andreoni and Colton, 2017). Of the two main etiological agents that cause HFMD, only EV71 leads to neurological complications. (**Figure 1**; Komatsu et al., 1999; Wang et al., 1999; McMinn et al., 2001). Since the almost total eradication of poliovirus, EV71 has become the most important neurotropic *Enterovirus* in the world. However, there is still much that is unknown about the interactions of this virus and more studies are required in this area to fully unravel the complete mechanism of EV71 (Lin et al., 2002a; Huang and Shih, 2015).

Enterovirus 71 has been found all over the world with different symptoms and clinical manifestations seen during different outbreaks. The virus was first isolated in California, United States in 1969 from patients suffering from central nervous system (CNS) infections with cutaneous signs (Schmidt et al., 1974). EV71 was next seen in Europe, in Bulgaria and Hungary where patients suffered from CNS infections with cardiorespiratory failure and acute flaccid paralysis (Chumakov et al., 1979; Nagy et al., 1982). EV71 was also starting too been seen in Asia at this time, with Japan reporting outbreaks of HFMD and CNS infections caused by EV71 (Hagiwara et al., 1978; Ishimaru et al., 1980). In 1997 an outbreak of EV71 presenting with HFMD, cutaneous infections, CNS infections, cardiorespiratory collapse and sudden death was reported in Malaysia (Lum et al., 1998; Chan et al., 2000). Since then, outbreaks and epidemics of EV71 with these clinical manifestations have become endemic to the Asian–Pacific region (Ho et al., 1999; Komatsu et al., 1999; McMinn et al., 1999; Fujimoto et al., 2002; Lin et al., 2003b; Tu et al., 2007; Sazaly et al., 2009; Kim, 2010; Wu et al., 2010; Tan et al., 2011).

This protracted epidemic has drawn specific scientific interest. These outbreaks, with their larger variety of symptoms have drawn more questions that need answering about EV71 and its pathophysiology.

## Biology of EV71

### Genome Organization

**FIGURE 2:**
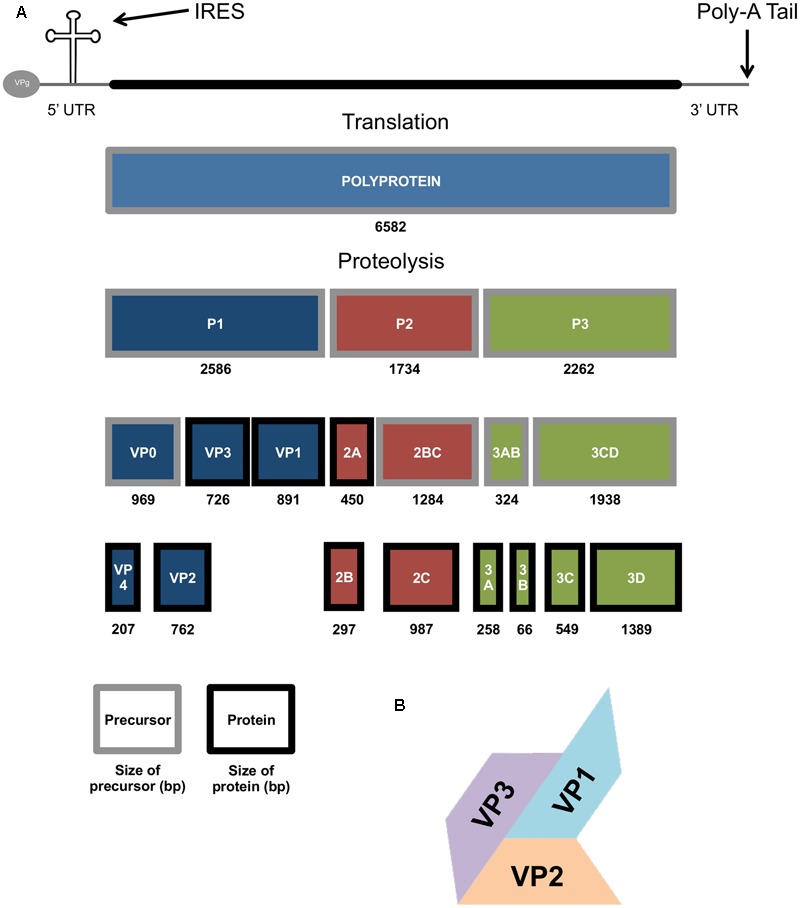
The genomic and molecular make up of the EV71 genome. **(A)** Diagram showing the EV71 genome, polyprotein and protein products produced during EV71 replication, size in base pairs (bp) also show. **(B)** Picture representation of the *T* = 3 structure of the VP1-3 structural genes of the EV71 capsid.

Enterovirus 71 is a positive sense RNA virus, with a genome approximately 7.4 kb long with one open reading frame coding for the 4 structural (VP1-4) and 7 non-structural proteins (2A-C and 3A-D) (Brown and Pallansch, 1995). The genome is initially translated as a single polyprotein, which is then cleaved into the three cleavage intermediates P1, P2, and P3 (**Figure 2**). P1 is cleaved into VP3, VP1, and VP0 (which is then in turn cleaved to form VP4 and VP2). The VP1, VP2, and VP3 proteins form a surface pentameric subunit together with the VP4 protein, attached to the inner surface (**Figure 2**). The capsid is formed by a quasi-*T* = 3 symmetry of 60 copies of this subunit to form an icosahedral capsid (Plevka et al., 2012). P2 is cleaved to form the viral protease 2A, and the 2BC polyprotein, which is then further cleaved in to the two non-structural proteins 2B and 2C. P3 is initially cleaved into 3AB and 3CD, and then further proteolysed to form the 3A, 3B, 3C, and 3D proteins (**Figure 2**; McMinn, 2002).

EV71 has been divided in to 6 genogroups, A, B, C, D, E, and F. The genogroups B and C can be further divided into the sub-genogroups B0–B5 and C1–C5. These genotypes were identified through genetic analysis of the VP1 gene (Tee et al., 2010). All of the genogroups of EV71 have an amino acid (aa) sequence similarity of over 90% to the virus genogroup A prototype BrCr strain; with *B* > 92.2%; *C* > 91.9%; *D* = 95.2%; *E* > 93.6% and *F* > 93.6% (Bessaud et al., 2014). They also have a high amino acid sequence similarity within the subgenogroups individually, with *A* > 94.9%; *B* > 95.9%; *C* > 94.2%; *E* = 96.9% and *F* = 96.9% (information on D not available) (Bessaud et al., 2014). While the B and C subgroups have a global distribution, D, and E and F are restricted to India and Africa, respectively (Bessaud et al., 2014). Further phylogenetic analysis and Bayesian relaxed molecular clock method experiments suggests than EV71 originally emerged from CA16 around 1941 (Tee et al., 2010).

### Virus Life Cycle

As humans are the only known hosts of EV71, all transmission is assumed to be human-to-human. The virus can survive on external surfaces for up to 3 days, so direct human-to-human contact is not always necessary as the virus can be picked up from these contaminated areas (Pallansch and Roos, 2001). The main route of transmission is through the fecal-oral route (Pallansch and Roos, 2001), however, it can also be spread through contact with virally contaminated vesicular fluid, surfaces, fomites and oral secretions as well as respiratory droplets (**Figure 1**; Solomon et al., 2010). EV71 has been also found in fecal samples of symptomatic patients up to 75 days after infection, and up to 14 days in the throat (Chung et al., 2001).

Evidence suggests that the initial phase of replication occurs in lymphoid tissues of the tonsillar crypt (He et al., 2014) and in the Peyer’s patches of the small intestine (**Figure 1**; Pallansch and Roos, 2001). The virus undergoes further replication in the adjacent lymph nodes (cervical and mesenteric lymph nodes, respectively) and this often leads to a mild viremia (Cheng et al., 2014). In the majority of patients, viral infection is controlled at this point and the patients remain asymptomatic (Lee M.-S. et al., 2012). However, if the viral infection is not controlled, the virus will then reaches high titres, and disseminate to the skin and mucous membranes to cause HFMD. In a small proportion of patients, EV71 can also enter the CNS and cause severe complications (**Figure 1**; Solomon et al., 2010).

It has been noted that children who had detectable viraemia after 3 days had significantly more severe complications (**Figure 1**; Cheng et al., 2014). Retrograde axonal transport has been proposed as the method of entry to the brain and CNS for EV71, as when mice were inoculated at the hind limbs with EV71 the virus appeared to be spread from the lower to the upper spine (Chen et al., 2007). However, with many receptors for EV71, including one on lymphocytes (Nishimura et al., 2009) retrograde axonal transport may not be the only mode of entry. After gaining entry to the brain and CNS, EV71 causes neuronal and astrocytic cell death (**Figure 1**; Chang et al., 2004, 2015), which in turn leads to the CNS immune and inflammatory response and brainstem encephalitis (**Figure 1**; Chang et al., 2015). This leads to the mass release of certain cytokines, known as a cytokine storm (Lin et al., 2003a). There have been many cytokines implicated in EV71 brainstem encephalitis which have been reported to be significantly increased in patients that suffer from pulmonary oedema. These include IL-1β, IL-1RA, G-CSF (Griffiths et al., 2012), IL-6, IL-10, TNF-αα and IFN-γ (**Figure 1**; Lin et al., 2002b, 2003a; Ye et al., 2015). The destruction of the brainstem during EV71 infection, including the vasomotor and respiratory centers leads to a surge of catecholamines and autonomic dysfunction (Wang et al., 2003; Kao et al., 2004), which may be the cause of cardiorespiratory problems (**Figure 1**). It is currently unknown what is the exact cause of cardiac failure and pulmonary oedema but is thought to be a combination of these elements, cytokine storm and brainstem destruction.

### Host Receptors

At least five molecules have been identified as possible cell surface receptors for EV71. Scavenger receptor B2 (SCARB2) (Yamayoshi et al., 2009), P-selectin glycoprotein ligand-1 (PSGL-1) (Nishimura et al., 2009), sialylated glycan (Yang et al., 2009), heparan sulfate (Tan et al., 2013) and annexin II (Anx2) (Yang et al., 2011) have been reported to act, individually, as receptors for EV71. The most characterized of these five are scavenger receptor class B member 2 (SCARB2) and PSGL-1 (Nishimura et al., 2009; Yamayoshi et al., 2014). SCARB2 (also known as LIMP-2) is a highly abundant protein found in the lysosomal membrane, which participates in the reorganization and membrane transport of the endosomal/lysosomal compartment. SCARB2 can acts as a receptor for all strains of EV71 and is considered the critical receptor for infection (Yamayoshi and Koike, 2011). PSGL-1, which is primarily expressed on the surface of leukocytes, is involved in leukocyte rolling/interacting with the vascular endothelium in the early stages of inflammation (Hirata et al., 2000). A post translational modification, namely the tyrosine sulphation at the N-terminus of PSGL-1 was identified as being crucial for the binding of PSGL-1 to EV71 and for viral replication in lymphocytes (Nishimura et al., 2010). Some EV71 subgroups do not utilize PSGL-1 as a receptor to enter immune cells, as they cannot bind to the receptor. Only viruses with a G or Q amino acid at residue 145 of the structural protein VP1 can bind to PSGL-1 (Kataoka et al., 2015).

Mouse L929 cells that normally do not support EV71 infections, became highly infected with EV71 after being transfected and overexpressed with either SCARB2 or PSGL-1 (Yamayoshi and Koike, 2011). Although the L-SCARB2 cells were shown to be more susceptible to EV71 infection than L-PSGL-1 cells, they bound with a lower amount of EV71 when compared with the L-PSGL-1 cells. This indicates that the binding ability of the receptor does not determine the infection efficiency of said receptor (Yamayoshi and Koike, 2011).

## Infection and Immunity

As there is currently no effective treatment available for EV71, understanding virus–host interactions in greater detail will provide an increased knowledge base for the successful development of medical countermeasures (Ooi et al., 2010).

The innate immune response is one of the body’s earliest ways of fighting off pathogens (Takeuchi and Akira, 2009). This immune response uses specific receptors that can recognize diverse foreign moieties and then launch an immune response to try and limit the infection (Takeuchi and Akira, 2009). The innate immune response is much less specific than the adaptive immune response, but much more rapid and reacts instantaneously upon the discovery of pathogens. The first line of defense contributes by effectively limiting the infectivity of a pathogen, as well as activating the adaptive immune response to help destroy and clear the pathogenic organism (Takeuchi and Akira, 2009).

Host cells, both immune and non-immune contain innate receptors called pattern-recognition receptors (PRRs), which can recognize materials/molecules of pathogens, and components of cell damage/death. These PRRs recognize the pathogens via the detection of pathogen associated molecular patterns (PAMPS) (Mogensen, 2009). PRRs can also recognize molecules from stressed/dying cells, named danger associated molecular patterns (DAMPs) and launch a response (Tang et al., 2012). The main receptors of viral PAMPs are toll-like receptors (TLRs), nucleotide oligomerization domain (NOD) like receptors (NLRs) and retinoic acid inducible gene I (RIG-I) like receptors (RLRs) (Fukata et al., 2009).

### Innate Immunity against EV71

Enterovirus 71 is mainly detected via the RLRs; RIG-I, melanoma differentiation-associated protein 5 (MDA5), and TLR3 (Pathinayake et al., 2015). The binding of EV71 to the RLRs sets off a signaling cascade activating mitochondrial antiviral-signalling protein (MAVS). MAVS associates with tumour necrosis factor (TNF) receptor associated factor 3 (TRAF3), recruiting TANK-binding kinase 1 (TKB1) and IκB kinase, leading to the phosphorylation of interferon regulatory factors 3 and 7 (IRF-3 and IRF-7) (Fitzgerald et al., 2003). This phosphorylation causes these two IRF molecules to dimerise, causing the formation of hetero- and homodimers, which translocate to the nucleus and bind to interferon stimulate response elements (ISREs). This leads to the expression of type I interferon genes (Fitzgerald et al., 2003). MAVS activates NF-κB through a caspase 8/10 dependent pathway (Wang B. et al., 2013). TLR3 also activates NF-κB by inducing Interferon- β (IFN-β), which associates with receptor-interacting protein 1 (RIP1), activating NF-κB. This coordinated network activates many pro-inflammatory cytokines, chemokines, enzymes, and adhesion molecules to fight the pathogen (Lei et al., 2011).

Interferons (IFN’s) are molecules that are vital to the body’s immune response in order to help control virus replication and spread (Kontsek, 1994). Based on the interaction they have with their receptors, they have been categorized into three groups; type I, type II, and type III. Both type I and type III IFNs have been suggested to play vital roles in the defense against viral infections (Reid and Charleston, 2014). Specific interferon-α (IFN-α; a type I IFN molecule produced by leukocytes) subtypes have been shown to be inhibitors of EV71 infection (IFN-α4, 6, 14, and 16) (Yi et al., 2011). Unfortunately, IFNs have only been shown to be effective as pre-treatments as *in vivo* and patient studies suggest that IFN’s offer little help as a treatment after contraction of the virus (Liu et al., 2005).

Viral proteases are vital for the processing of most viral polyproteins. EV71 relies on its two proteases, 2A and 3C, to cleave viral protein precursors into their functional forms (**Figure 2**; Lin et al., 2009a). These proteases not only cleave viral proteins, they can also cleave host proteins to aid in immune evasion (Shih et al., 2011). Viral protease 2A has been shown to cleave MDA5, the main cellular receptor for detecting EV71 infection (**Table 1**). This cleavage diminishes the production and activation of IRF3 and IFN type I (Feng et al., 2014). The EV71 protease 2A can also cleave MAVS at several sites which again leads to the disruption of IRF3 phosphorylation and a decrease in type I IFN production, leading to increased EV71 propagation, compared to if the response was active (Wang B. et al., 2013). Data has been presented that suggests 2A interferes with IFNAR I by reducing its expression (Lu et al., 2012). The reduction in IFNAR I expression has been postulated as why exogenous IFN has had limited effect as a treatment (Morrison and Racaniello, 2009). However, this hypothesis is still contested by some (Liu Y. et al., 2014), and more evidence will be needed to address this hypothesis.

**Table 1:** Table showing the reported functions of the individual EV71 proteins on virus and host.

Protein	Function
VP1-3	Structural proteins, form icosahedral capsid. 60 copies of pseudo *T* = 3 symmetry
VP4	Structural protein, lies on the inside of the capsid, implicated in viral genome release
2A	A protease, important for viral polyprotein and host protein cleavage
2B	A viroporin, creates pores in the host ER
2C	Induces structural rearrangements, not too much known about this protein.
3A	Localizes viral replication complexes to vesicle membrane surfaces
3B	Viral protein genome-linked (VPg)
3C	A protease, cleaves viral precursor proteins as well as host proteins such as retinoic acid inducible gene I
3D	RNA Polymerase, replicates viral RNA

Inhibition of the host interferon response has also been shown by the 3C protease (Lei et al., 2010). 3C interacts with the N-terminal domain of RIG-I preventing the interaction with MAVS and leading to a decrease in IRF3 and IFN (**Table 1**; Lei et al., 2010). Similarly EV71 3C has been shown to bind and degrade IRF 9 (Hung et al., 2011) and to cleave IRF 7 (Lei et al., 2013) impairing the ability of these two cellular proteins to stimulate the production of ISGs and IFN.

### EV71 and Host Replication Factors

The genome of EV71 is uncapped and therefore initiation of translation by ribosomes are facilitated by the presence of a type I internal ribosome entry site (IRES) (Lin et al., 2009b). This tertiary RNA structure is a common feature at the 5′ end of picornaviruses. Initiation of translation at an IRES requires specific cellular proteins and their absence can lead to inefficient IRES-dependent translation. During EV71 and general picornavirus infection, host cell factors, known as IRES transacting factors (ITAFs), involved in the initiation of cap-dependent translation on host mRNAs are degraded by viral proteases (Shih et al., 2011). Thus, inhibiting cap-dependent host cell translation and facilitating the translation of viral genomes (which act as an mRNA) (Pilipenko et al., 2001). During viral infection, EV71 expresses viral protease 2A that cleaves eIF4G, part of the eukaryotic initiation factor 4F complex (eIF4F) needed for cap dependent translation. Although these results in a significant decrease in the hosts capped mRNA translation, it increases the translation efficiency of the EV71 IRES (**Table 1**; Thompson and Sarnow, 2003).

Some novel ITAFs for EV71 have also been discovered. Far upstream element binding protein 1 and 2 (FBP1 and 2) have both been identified as novel ITAFs for EV71 (Huang et al., 2011; Chen et al., 2013). FBP1 enhances the replication activity of the virus and IRES-dependent translation. FBP2, however, has been shown to be a negative regulator of IRES activity, by out competing the positive ITAF hnRNP1 (Lin et al., 2009b). However, EV71 infection triggers proteasomal, autophagic, and caspase activity mechanisms of the host cell, which cleave FBP2, and these cleavage fragments transform FBP2’s function from a negative regulator to a positive promoter (**Table 1**; Huang et al., 2011; Chen et al., 2013).

Enterovirus 71’s ability to interrupt, intercept and disrupt the host immune response is critical to the survival and propagation of the virus, and whilst this has a negative effect for the patients, it gives an insight into possible areas that could be targeted to disrupt virus biology.

## Deciphering Pathophysiology Using *in Vitro* and *in Vivo* Models

### *In Vitro* Cellular Models

*In vitro* models whilst being the initial “go to” model for viral experiments, can come in many different forms and serve many different purposes. The most basic of these are isolation and viral kinetics, for which rhabdomyosarcoma (RD) cells are the most utilized for EV71 (Pérez-Ruiz et al., 2003). One major advantage of *in vitro* cellular models is that they can be adapted and modified very easily. This aids the discovery of important proteins and cellular functions that would otherwise be extremely difficult. The EV71 cellular receptor, SCARB2 was identified utilizing *in vitro* techniques. Mouse L929 cells, not known to be targeted by EV71 were first transfected with RD cell genomic DNA and then infected with EV71. Transcriptomics analysis on highly infected cells identified SCARB2 as the receptor responsible for this new ability to become infected (Yamayoshi et al., 2009). PSGL-1 was discovered using Jurkat cells, an immortalized T cell line (Nishimura et al., 2009). A retroviral cDNA library from EV71 susceptible Jurkat cells was generated and then used for expression cloning. Transduction of P3U1 produced four colonies that bound to EV71 coated dishes, which all encoded PSGL-1 (Nishimura et al., 2009). Without this *in vitro* model, this discovery may not have occurred as *ex vivo* primary T cells do not proliferate, and therefore this type of experiment would not have been feasible.

Another advantage of using cell lines is they can provide lines that are not always readily available as primary cells. Primary human monocytes and T cells often not easily accessible due to availability or ethics, however, with *in vitro* cell lines such as THP-1 (monocytes) or Jurkat (T cells) these issues can be easily over come. THP-1 cells have been used to show the role of NLRP3 inflammasome activation and IL-1β maturation during interaction with the EV71 protein 3D (Wang et al., 2017).

### *Ex Vivo* Models

Whilst they are not always readily accessible, primary *ex vivo* models can be of huge benefit to investigations as they bridge the gap between immortalized *in vitro* cell lines and *in vivo* animal models. They provide insights into how the body might react to an infection without any chance of harm to the host. Understanding EV71’s ability to infect immune cells and their possible role in EV71 severity and fatalities was not always clear. However, using *ex vivo* models allowed studies to investigate the direct effect EV71 has on these immune cells and any indirect downstream effects (Chen and Yeh, 2009). EV71 was shown to directly infect monocytes and lymphocytes from freshly isolated blood mononuclear cells (PBMCs) and induce the production of proinflammatory cytokines such as TNF-α and MIF during the course of an infection (Chen and Yeh, 2009). *Ex vivo* models have been used to show how the activation of JNK1/2 and p38 MAPK pathways can promote EV71 infection in dendritic cells (DCs) (Peng et al., 2014).

### *In Vivo* Animal Models

Interestingly, *in vivo* mouse models of EV71 have demonstrated an age-dependent susceptibility to the virus (Arita et al., 2008; Chua et al., 2008; Khong et al., 2012; Fujii et al., 2013). Non-transgenic, immunocompetent mice are resistant to EV71 infection once they are past the weaning stage, regardless of the route of infection (Arita et al., 2008; Chua et al., 2008; Khong et al., 2012; Fujii et al., 2013). In an effort to develop a EV71 mouse model more similar to the onset of human disease, mouse-adapted EV71 strains have been generated that are capable of infecting mice via the oral route, and can cause neuropathology in the brainstem and spinal cord as well as paralysis of the rear limbs (Chen et al., 2004; Wang et al., 2004; Ong et al., 2008). However, these models still have an age limitation and do not cause the onset of disease after the mice reach 14 days old (Chen et al., 2004; Wang et al., 2004; Ong et al., 2008). Whilst these models are not ideal due to their age-limiting factor, they have still proven to be useful tools in the examination of EV71 pathogenesis. This model was used in the discovery of type I interferon being an essential innate defense mechanism in the controlling or EV71 infection and that EV71 tries to avoid this mechanism by inhibiting type I interferon through its 3C protease (Liu et al., 2005; Lee Y.P. et al., 2012).

To get around this age limitation, immunocompromised models such as NOD/SCID (Arita et al., 2008) and AG129 (Khong et al., 2012) mice have been used which cause similar features and symptoms seen in the immunocompetent mouse models but in older mice (Arita et al., 2008; Khong et al., 2012). Whilst this is an advantage, it also has its drawbacks, namely immunocompromised mice stray further from the human model they are trying to replicate and thus give us a less reliable report on what may be causing these symptoms in humans (Shultz et al., 2012). Although a more recent study could replicate skin rash induced by EV71 infection in the NOD/SCID mouse model (Liao et al., 2014).

Transgenic mice containing the human EV71 receptors SCARB2 and PSGL-1 have been generated and been shown to be infected with EV71 to varying degrees (Liu et al., 2012; Fujii et al., 2013). Mice expressing the PSGL-1 gene are only susceptible to mouse-adapted strains of EV71, and whilst this model exhibited symptoms that were similar to those seen in wild-type mice, these mice suggest that PSGL-1 cannot provoke EV71 infectivity in mice on its own (Liu et al., 2012). Transgenic mice that express the human SCARB2 in their CNS neurons, lung pneumocytes, hepatocytes, and intestinal epithelium (i.e., similar profile to humans) have been shown to be susceptible to infection by EV71 after they are 6 weeks of age. These mice display symptoms of neurotropism, neuropathology, ataxia, paralysis, and death, which are similar to symptoms seen in humans (Fujii et al., 2013).

To try and replicate the human disease model, mice have been orally infected with EV71 with differing degrees of effectiveness. Non-adapted virus strains have been shown to cause skin lesions in immunocompetent neonatal mice, but other than this symptom, animals typically continued to grow normally with no neurological effects (Chen et al., 2004). However, mouse-adapted strains have been shown to not only cause skin lesions but also movement disorientation, hind limb paralysis followed by death (Chen et al., 2004). When using immunocompromised mice, orally infected non-adapted virus was capable of causing neurological complications and death (Khong et al., 2012; Liao et al., 2014). Whilst this route is much closer to the natural route of infection then intra-spinal, cranial or peritoneal injections, these are still very young mice and do not represent the same respective age that the disease affects humans. These mice are still fed by their mothers, at this stage in human development children are still protected by maternal antibodies passed through passive transfer (Hogarth, 1982) and breast milk (Hanson, 1998), therefore much less susceptible to the disease.

Non-human primates have been shown to be susceptible to EV71 infection and have since been used in various studies (Nagata et al., 2002, 2004). Cynomolgus monkeys show very similar neurological manifestations of symptoms when infected intraspinally or intravenously, including acute flaccid paralysis, ataxia and encephalitis. The animals also presented with a broad viral circulation including spinal cord, cerebrum, brainstem, and CNS (Nagata et al., 2002, 2004). However, they do not suffer from blisters or lesions on the skin, nor do they suffer from pulmonary oedema (Nagata et al., 2002, 2004). A Rhesus monkey model has also been used to assess EV71 infection. These monkeys also develop CNS infections after intracranial, intravenous, intratracheal or orally given virus. Tissue damage and cellular infiltrates in the lungs were noted in these monkeys, they were not observed in the spleen or pancreas, which also show high viral load. Although these monkeys did not show vesicular lesions on the skin or typical neurological symptoms, half of them did suffer from pulmonary oedema when infected intracranially (Zhang et al., 2011). This is the first model to show this symptom outside of humans, however, it is not known if the pulmonary oedema is due to CNS damage and inflammation or if it is a consequence of viral cytolysis in the lungs (Zhang et al., 2011).

Whilst there are many advantages that come with using animal models, there are also some drawbacks. One issue is the use of animal-adapted viruses. EV71 mouse models are very limited by the age of the mouse. To circumvent this, mouse-adapted EV71 strains are used. As EV71 is a positive sense single stranded RNA virus it can evolve quickly through mutations due to the lack of proofreading mechanisms (Drake and Holland, 1999). All viruses will contain quasi-species that will continue to be introduced with multiple passages during animal species adaptation. These mutations can cause significant differences to the virus, such as structural changes (Chua et al., 2008) and alterations in the host–virus interactions that differ from the wild-type strain (Chua et al., 2008). The mutation of G145E in the VP1 protein of EV71 has been shown to increase the level of morbidity and mortality during EV71 infection in mice (Chua et al., 2008; Huang et al., 2012).

Whilst these animal models are not perfect representations of the disease or the route it takes in humans, they still provide us with lots of crucial information on the role of the virus and of the host immune response. Notably, a significant study recently reported the generation of a hybrid mouse model by cross-breeding between STAT-1 KO and hSCARB2 transgenic mice. This model was able to support efficient lower titer (1 million pfu/mouse) EV71 experimental infection using older mice. Therefore, this hybrid model lends a strong support to the importance of both entry factor and host innate immunity (Liou et al., 2016).

## Potential Therapeutics for EV71

### Antivirals

Many drugs that have been already developed and used to fight other picornaviruses, such as polioviruses and rhinoviruses, were tested for their abilities to limit EV71 infection. These drugs, ribavirin (Li et al., 2008; Zhang et al., 2012), pleconaril (Rotbart and Webster, 2001; Zhang et al., 2012) and rupintrivir (Zhang et al., 2013) have all shown some sort of protective antiviral effect in mouse models. However, it remains to be seen how this would be translated to human infections. Also as the EV71 genome is synthesized through the virus’s 3D polymerase (Liu Y.-C. et al., 2014), which lacks a proofreading activity, mutations frequently arise and could quickly lead to the generation of antiviral resistant viruses (Chen et al., 2009).

The recent discovery of the crystal structure of EV71 (Plevka et al., 2012) has shed some insights into possible drug targets for future antivirals against EV71. The resolution of this structure by x-ray crystallography showed the presence of a hydrophobic pocket factor located underneath a depression known as the canyon. It has been postulated that the binding of molecules to this pocket region could stabilize the capsid and therefore inhibit the uncoating process induced by the EV71 receptor (Plevka et al., 2012). Several compounds have been created which bind to this pocket region and showed effective *in vitro* inhibition of EV7 (Shia et al., 2002; Chern et al., 2004; Shih et al., 2004a; Chang et al., 2005; Chen et al., 2008; Plevka et al., 2013). However, just a single point mutation was enough to confer resistance against this type of compound (Shih et al., 2004b).

Type I IFN, has been shown as an effective antiviral to treat viral infections such as HCV (Rong and Perelson, 2010), so experiments were done to investigate IFNs ability as an EV71 therapeutic. Studies have shown in both *in vivo* (Liu et al., 2005) and *in vitro* (Yi et al., 2011) systems that IFN could increase the survival rate and reduce EV71 replication (Liu et al., 2005; Yi et al., 2011). However, the viral proteases 2A and 3C have the ability to degrade IFN and the IFN antiviral pathway and may lead to the reduction in expression of interferon-α/β receptor (IFNAR) (Morrison and Racaniello, 2009; Lei et al., 2010, 2013; Wang B. et al., 2013; Feng et al., 2014). There have been reports of synergistic effects when 3C inhibitors and IFN are combined (Hung et al., 2011), but this would have to be combined with a 2A inhibitor to further inhibit the interruption and degradation of IFN.

### Monoclonal Antibodies

As there is no current antiviral treatment, and vaccine development, whilst currently underway, is not fully approved outside of China, human IVIG has been used on a presumptive basis as a last resort (Chea et al., 2015). This treatment has perceived positive benefits, possibly through modulatory properties or viral neutralization, but is not wholly effective and has not been tested via a randomized controlled trial. IVIG also poses a potential risk of infection via other pathogens. Humanized mouse monoclonal antibodies eliminate this risk whilst increasing the specificity against the virus. Humanized mouse monoclonal antibodies against other viruses such as RSV have already been approved by the FDA (The IMpact-RSV Study Group, 1998; Fenton et al., 2004).

There have been several studies that suggest that monoclonal antibodies could be an effective treatment against EV71 infection, and multiple sites on the virus have been found as possible targets. Plevka et al. (2014) raised two antibodies against an empty immature EV71 particle in mice. The structure of this particle, which is similar to the “A” particle seen when EV71 recognizes a host cell before genome release, differs from that of a mature virus. These antibodies were shown to neutralize EV71 *in vitro* by instigating a conformational change after incubation with the mature virus, transforming the infectious virus into an “A” particle and instigating genome release (Plevka et al., 2014).

Another antibody, produced against the EV71 virus like particle (VLP) showed potent neutralizing capabilities *in vitro* (Ku et al., 2012). It was later discovered that the antibody bivalently bound across the twofold axis of the EV71 virion, potentially preventing the conformational changes in viral capsid proteins required for viral genome release (Ye et al., 2016).

Antibodies raised in mice against a conserved VP3 knob region have shown protective neutralizing effect across the different subgroups of EV71 (Kiener et al., 2014). Whilst both antivirals and monoclonal antibodies remain important tools to fight EV71 infection, a vaccine still serves as our best chance at eradicating this virus, as shown with the almost total eradication of polio after the introduction of the Salk and Sabin vaccines (Pliaka et al., 2012).

### Vaccines

After the success of the polio vaccines, it was envisioned that EV71, closely related to polio, would also be an easy target for near eradication via a vaccine. Studies in mice demonstrated that the transfer of antiserum provided protection against EV71, further indicating the attainability of a vaccine (Yu et al., 2000). EV71 vaccine candidates have been suggested in many different forms; from attenuated strains (Arita et al., 2007), inactivated whole virus (Zhu et al., 2013b), and VLPs (Chung et al., 2008) to recombinant proteins (Wang M. et al., 2013) and peptide vaccines (Foo et al., 2007).

Live attenuated vaccines have been tested in cynomolgus monkeys and these induced the production of high levels of neutralizing antibodies that provided cross protection across the sub groups (Meng and Kwang, 2014). However, as with all live attenuated vaccines, there is the possibility for the virus to mutate and cause disease. One study showed a vaccine candidate producing mild neurological symptoms and being neurotropic when injected intravenously (Arita et al., 2007).

DNA vaccines have been attempted for EV71. However, DNA constructs containing EV71 VP1 gene that elicited specific VP1 immunoglobulin G’s (IgG’s) and neutralizing antibodies conferred low levels of antigenicity (Tung et al., 2007).

Inactivated whole virus vaccines have been shown in mouse models of EV71 to produce very high levels of virus specific antibodies that are cross neutralizing (Wu et al., 2001; Ong et al., 2010). These successful pre-clinical studies lead the way for phase I (Li et al., 2012) and phase II (Zhu et al., 2014) clinical trials, and since then three vaccines have moved on to phase III clinical trials, all from China and based on the C4 strain (Zhu et al., 2013a, 2014; Li et al., 2014). These trials involved over 30,000 children and resulted in the prevention of 90% of HFMD and 80% of other EV71 related symptoms, and the Chinese FDA has approved two of these vaccines. Although these vaccines have only been approved in China, this is a very exciting prospect in the eradication of EV71. However, there is still work to be done on antiviral treatment and therapies for the treatment of patients and to help unravel the mechanisms behind this disease.

## Conclusion

There have been major breakthroughs in EV71 research in the last couple of decades: from the identification of host receptors, to the discovery of molecular mechanisms of virus–host interactions, to the phase III trials for vaccines. Although there is an understanding of EV71 pathogenesis, many areas of uncertainty remain with many mechanisms still not well understood and many pathways that have yet to be proven functionally. There is still a need for an animal model that better mimics the human disease. The development of such models will enable us to gain a much better understanding of the path of EV71 infection, and may give us an insight in to new molecular mechanism that we can target to interrupt the virus and stop the infection.

## Author Contributions

JC searched the literature and compiled and wrote the review. LN helped design, write, and edit the review. JH, TS, and M-HO helped to write, edit, and critique the review.

## Conflict of Interest Statement

The authors declare that the research was conducted in the absence of any commercial or financial relationships that could be construed as a potential conflict of interest.
